# Development of artificial intelligence for automated measurement of cervical lordosis on lateral radiographs

**DOI:** 10.1038/s41598-022-19914-x

**Published:** 2022-09-21

**Authors:** Takahito Fujimori, Yuki Suzuki, Shota Takenaka, Kosuke Kita, Yuya Kanie, Takashi Kaito, Yuichiro Ukon, Tadashi Watabe, Nozomu Nakajima, Shoji Kido, Seiji Okada

**Affiliations:** 1 Department of Orthopedic Surgery, Graduate School of Medicine, Osaka University, 2-2 Yamadaoka, Suita, Osaka 565-0871 Japan; 2 Department of Artificial Intelligence Diagnostic Radiology, Graduate School of Medicine, Osaka University, Suita, Osaka Japan; 3 Department of Nuclear Medicine and Tracer Kinetics, Graduate School of Medicine, Osaka University, Suita, Osaka Japan; 4 Department of Orthopedic Surgery, Japanese Red Cross Society Himeji Hospital, Himeji, Hyogo Japan

**Keywords:** Neurology, Mathematics and computing

## Abstract

Cervical sagittal alignment is an essential parameter for the evaluation of spine disorders. Manual measurement is time-consuming and burdensome to measurers. Artificial intelligence (AI) in the form of convolutional neural networks has begun to be used to measure x-rays. This study aimed to develop AI for automated measurement of lordosis on lateral cervical x-rays. We included 4546 cervical x-rays from 1674 patients. For all x-rays, the caudal endplates of C2 and C7 were labeled based on consensus among well-experienced spine surgeons, the data for which were used as ground truth. This ground truth was split into training data and test data, and the AI model learned the training data. The absolute error of the AI measurements relative to the ground truth for 4546 x-rays was determined by fivefold cross-validation. Additionally, the absolute error of AI measurements was compared with the error of other 2 surgeons’ measurements on 415 radiographs of 168 randomly selected patients. In fivefold cross-validation, the absolute error of the AI model was 3.3° in the average and 2.2° in the median. For comparison of other surgeons, the mean absolute error for measurement of 168 patients was 3.1° ± 3.4° for the AI model, 3.9° ± 3.4° for Surgeon 1, and 3.8° ± 4.7° for Surgeon 2. The AI model had a significantly smaller error than Surgeon 1 and Surgeon 2 (P = 0.002 and 0.036). This algorithm is available at (https://ykszk.github.io/c2c7demo/). The AI model measured cervical spine alignment with better accuracy than surgeons. AI can assist in routine medical care and can be helpful in research that measures large numbers of images. However, because of the large errors in rare cases such as highly deformed ones, AI may, in principle, be limited to assisting humans.

## Introduction

Cervical alignment, an important clinical parameter in spine disorders, is associated with deformity, myelopathy, adjacent-segment disease, horizontal gaze, and health-related quality of life^1–3^. Measuring cervical alignment in multiple positions is important in evaluating pathology and planning surgery^4^.

Historically, such measurements have been obtained by using a protractor on radiographs. In recent years, digital viewer measurements became more common^5^, but surgeons generally still had to obtain measurements manually. Obtaining the necessary measurements for many parameters before and after surgery for a large number of patients requires a great deal of labor^6^. In scoliosis, the accuracy of measurement has been verified so far. Human measurement error has been generally reported to be approximately 3° to 7°, and this value is believed to be similar for the cervical spine^7–9^. Artificial intelligence (AI) models using convolutional neural networks (CNNs) have excellent capabilities for image recognition^10–12^. Because they require relatively less preprocessing than other algorithms, and because they automatically learn to optimize filters, whereas traditional algorithms do so manually^13,14^, they may reduce the labor involved in measurement.

A recent study^15^ of CNNs showed that the standard errors for determining lumbar lordosis in scoliosis ranged from 2.7° to 11.5°. Other studies have reported a mean absolute error (MAE) ranging from 4.3° to 8.1° when AI is used to assess lumbar lordosis^16,17^. There is room for improvement in the accuracy of AI models that measure x-rays. As for literature about plain radiographs of the cervical spine, some researchers detect ossification of the posterior longitudinal ligament using CNNs^12,18,19^. However, there are no studies that automatically measure cervical spine alignment. Additionally, previous programs were not surgeon-friendly because they must be operated through a character user interface. We thus conducted a study with the aim of developing AI in automated measurement of the C2–C7 angle on cervical x-rays through a graphical user interface.

## Methods

This study was approved by our institution’s review board (Osaka University Hospital Ethics Review Committee. No.20304) and written informed consent was waived because of the retrospective design. The study was performed in accordance with approved guidelines and in compliance with the principles of the Declaration of Helsinki.

### Study participants

**Figure 1:**
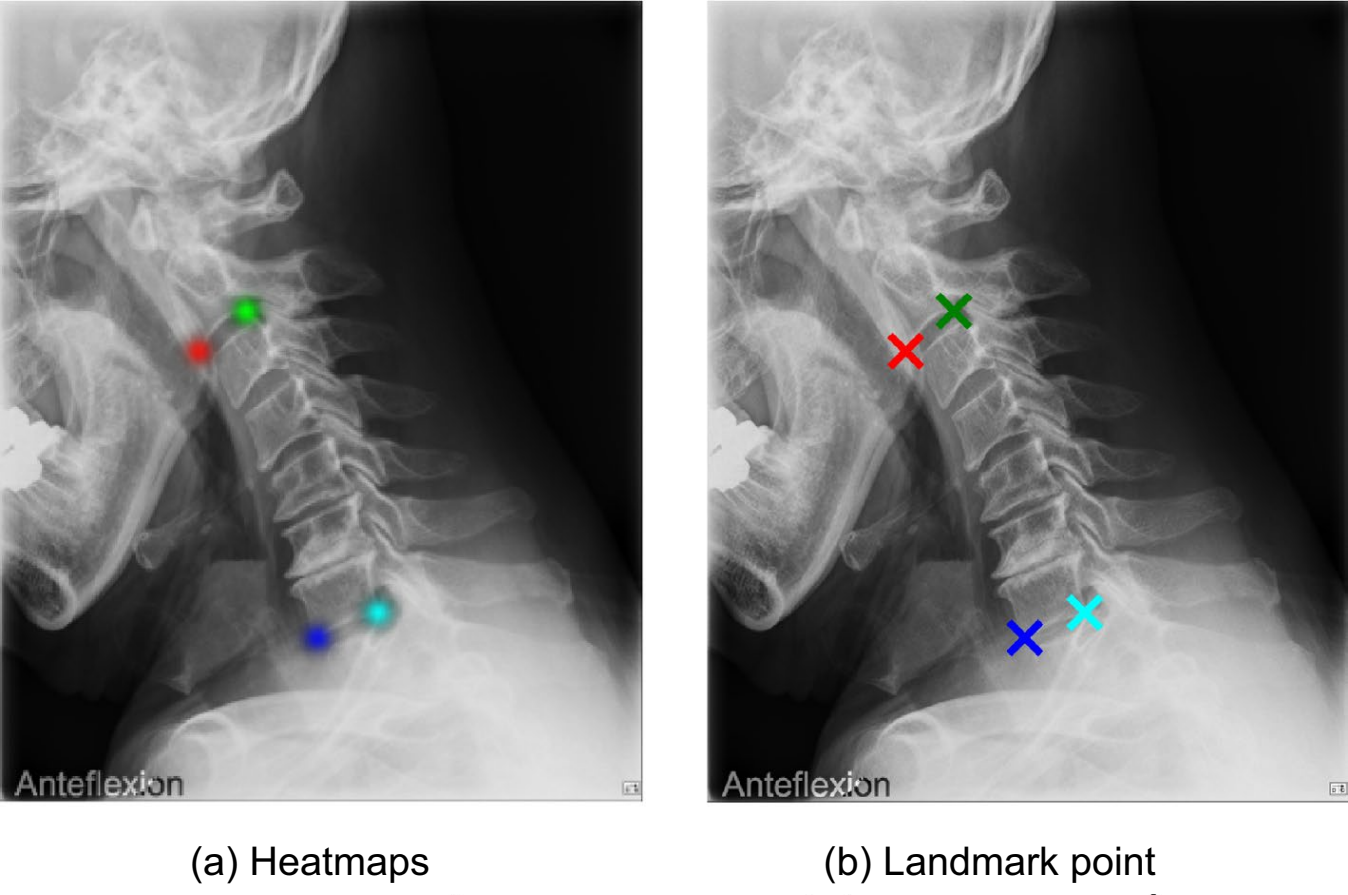
Most x-ray had a description of the posture in the corner of the x-ray. Anatomic landmark localization is done by extracting the coordinates with the maximum value from the heat map (**a**) of each landmark (**b**) output by the convolutional neural networks.

Study participants were surgical patients who underwent cervical spine surgery in our spine clinic between May 2012 and December 2020, and non-surgical patients who visited outpatient clinic between April 2019 and April 2021. Finally, 1674 patients with a total of 4546 x-rays were included in the study. To validate the capability of AI in real-world clinical practice, we did not exclude any patients who had deformities or who underwent spinal instrumentation, and all patients from the two lists were included in our study. All x-rays were measured on the lateral view and included flexion, extension, and the neutral position. Most of the x-rays had a description of the posture in the corner of them (Fig. 1). For x-rays that did not have a description, we could identify the position by comparing the x-rays with each other. X-rays were downloaded in DICOM (Digital Imaging and Communications in Medicine) file format and converted to PNG (Portable Network Graphic) file format.

### Method of radiographic measurement

**Figure 2:**
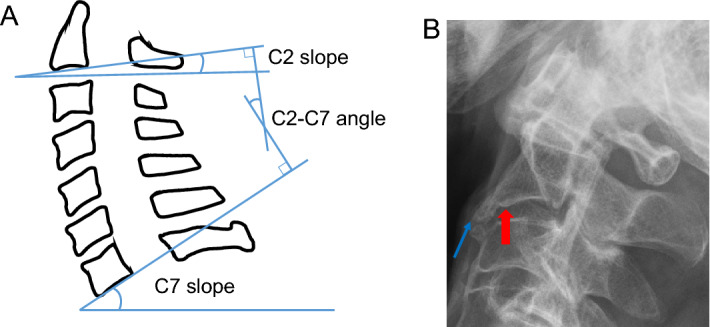
(**A**) The C2 slope is the angle between the C2 lower endplate and the horizontal line, and the C7 slope is the angle between the C7 lower endplate and the horizontal line. The C2-C7 angle is the angle between C2slope and C7slope. (**B**) The red arrow indicates the original vertebral corner. The blue arrow indicates the tip of the osteophyte. The point is marked at the red arrow.

We used the Cobb method to measure the C2–C7 angle because it is simple and most commonly used^1,20^. We labeled the anterior and posterior endpoints of the C2 inferior endplate as anatomic landmarks in a digital viewer to draw a straight line along the C2 inferior endplate, and we used the same method for the C7 vertebra (Fig. 2A). Because the endplate has a curve shape, the lowest point of the curve was marked as a rule. The exception was when an osteophyte was present. If an osteophyte was present at the corner of the vertebral body, we marked the original vertebral body corner, not the tip of the osteophyte (Fig. 2B). If the C7 vertebral body was obscured by the shoulder girdle and difficult to see, we used the C6 vertebral endplate as a reference for the C7 vertebral endplate. We used a publicly available image annotation software labelme (https://github.com/wkentaro/labelme) for this manual measurement process.

We labeled the C2 slope and the C7 slope, which are the angles that the C2 lower endplate and the C7 lower endplates make with the horizontal line, with clockwise being positive in both cases. The angle obtained by subtracting the C2 slope angle from the C7 slope angle is the C2–C7 angle, with a negative angle indicating lordosis and a positive angle indicating kyphosis.

### Artificial intelligence model

The AI model detected four anatomic landmarks: the anterior and posterior endpoints of the C2 and C7 inferior endplates. This anatomic landmark localization was performed by using CNNs to produce a heat map and then extracting the coordinates with the maximum value from the heat map of each landmark^21^ (Fig. 1).

For the CNNs to output heat maps, we used the DeepLabV3 segmentation architecture^22^, with the EfficientNet-B4^23^ as a backbone. DeepLabV3 is a segmentation architecture that uses atrous convolution to enlarge the field of view of the network and Efficient Net is a classification model that has eight variations with varying model sizes and accuracies. We chose EfficientNet-B4 for its good balance between the computational cost and accuracy. CNNs and angle measurements were implemented using Python version 3.9.5 (programming language) and PyTorch version 1.8.1 (an open-source machine learning framework). Our model was built using Segmentation Models Pytorch (https://github.com/qubvel/segmentation_models.pytorch), which is a publicly available package of Python and the backbone (EfficientNet-B4) was pretrained with ImageNet. The training of CNN was performed using Adam optimizer with initial learning rate of 0.001 using the root mean square as the loss function until the loss of the validation data extracted from the training data started to drop (i.e., just before overfitting). Initial learning rate was determined using a small subset (one tenth) of the entire dataset prior to the validation study. We used the largest possible batch size (, which was eight) that could fit in our workstation with 48 GB of total GPU memory.

The value on the heat map for each landmark was used as the confidence score, and the smallest of the four values was used as the confidence score for that x-ray. We used confidence scores for later analysis.

### Creation of ground truth data

**Figure 3:**
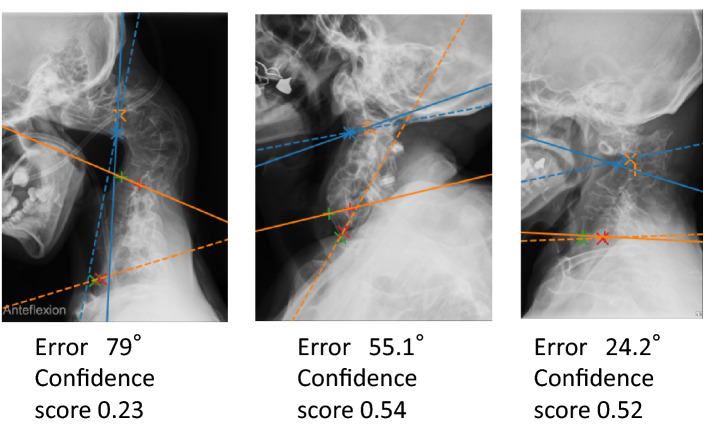
Three examples with measurement difficulties. In cases of fused vertebrae with a malformation, it is difficult for even surgeons to recognize the vertebrae correctly. The x-ray on the right is one of five cases in which two surgeons could not agree. The seemingly C2 vertebra was a malformed vertebra with multiple fused vertebrae. It is difficult even for surgeons to determine which part of this deformed vertebra is the original C2. Depending on which of the fused vertebrae is determined to be C2, the position of C7 will also change, which can easily lead to a large error. The solid lines represent the ground truth, and the dashed lines represent the measurement obtained by artificial intelligence.

In machine learning, ground truth is labeled data that are considered to be the correct values. A well-experienced spine surgeon with 18 years’ experience labeled the C2 and C7 endplates on all 4546 x-rays. Another spine surgeon with 20 years’ experience checked all these labeled points and proposed for correction for 123 x-rays. Agreement was reached between the two surgeons on 118 x-rays. For the remaining 5 x-rays that were not agreed on, we discussed with a surgeon with 23 years of experience and a surgeon with 37 years of experience, and a final agreement was reached. All these five X-rays were of cases with congenitally fused vertebrae at C2 (Fig. 3). Through these processes, we regarded these labeled 4546 x-rays as the ground truth.

### Validation of accuracy

**Figure 4:**
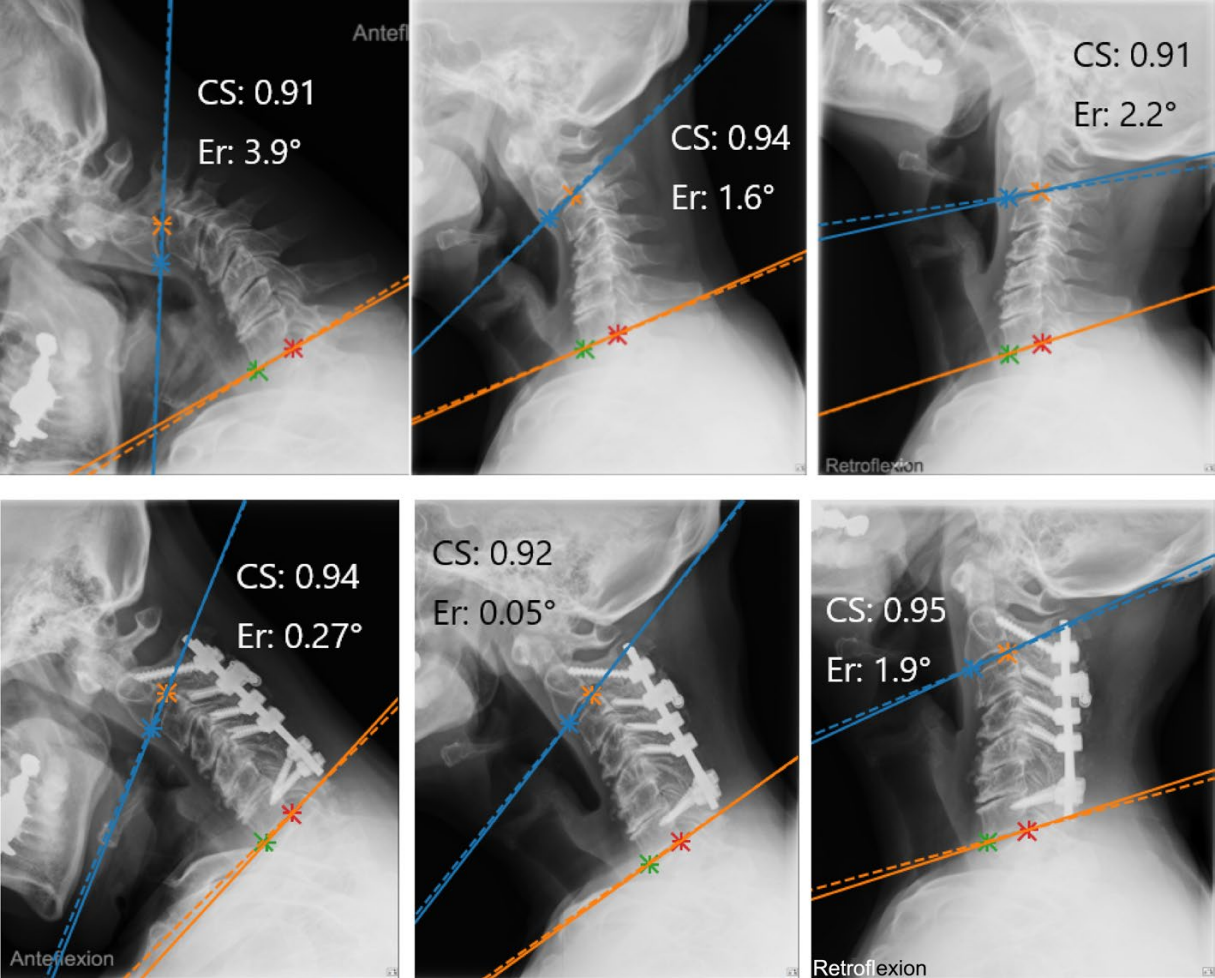
Preoperative (upper) and postoperative (lower) x-rays of a 54-year-old man. The solid lines represent the ground truth, and the dashed lines represent the measurement obtained by artificial intelligence. CS, confidence score; Er, error.

We examined measurement accuracy using two techniques. The first technique involved the absolute error of the AI algorithm’s measurements relative to the ground truth, calculated by fivefold cross-validation. We randomly divided all ground truth data into five groups: four groups were training data, and one group was test data. The algorithm learned the training data of the four groups and measured the test data of the remaining one group. We then calculated the absolute error of the algorithm’s measurements and the ground truth measurements on the test data (Fig. 4). This process was repeated five times, changing the training and test data groups so that all data were tested. Finally, the average of these absolute errors obtained from five processes represents the accuracy of the algorithm’s measurements. We did this five-grouping based on each patient, not based on each x-ray; it did not happen that the training data contained x-rays from one position, and the test data included x-rays from another position for the same patient. We performed validation on a workstation with two NVIDIA computers with GeForce RTX 3090 graphics-processing units, and the CNNs and angle measurements were implemented using Python (a programming language) and PyTorch (an open-source machine learning framework). The training of each CNN was performed until the accuracy of the validation data extracted from the training data dropped (i.e., just before overfitting).

The second technique involved comparing the accuracy of the AI algorithm’s measurements with that of other surgeons. Surgeon 1, with 12 years’ experience, and Surgeon 2, with 8 years’ experience, were both spine surgeons. From 1674 patients, we randomly selected 168 patients (57 men and 111 women) with a total of 416 x-rays, and each surgeon measured these according to the Cobb method described in the section “Method of Radiographic Measurement.” The surgeons were familiar with Cobb angle measurement method because the technique is standard. In addition, they were pre-trained on how to measure using labelme. The CNNs were trained on 1506 patients (4130 x-rays), excluding the 168 test patients, and measured on 168 patients (416 x-rays). We compared the error for the AI algorithm with the error for Surgeon 1 and for Surgeon 2.

### Setting the confidence score

We set the confidence score to measure the level of confidence in the measurements of the AI algorithm. The confidence score is expressed as a value between 0 and 1, where 0 indicates no confidence and 1 indicates confidence. By varying the confidence score as a threshold, we examined the relationship between the number of excluded x-rays and the absolute error.

### Relationship between the absolute error of artificial intelligence and background data on participants

We performed a multiple regression analysis with absolute error as the response variable and with age, sex, whether the patient had undergone surgery, and cervical spine position (flexion, neutral, and extension) as explanatory variables. The absolute errors were compared between the group of patients who had undergone surgery and the group of those who had not.

### Relationship between the absolute error and the number of training images

We tested how much the absolute error changes by increasing or decreasing the number of training images. We used 415 images (168 cases) that were used for comparison with other surgeons as test images. We randomly selected training images from the remaining 4131 images. We varied the training images to 200, 400, 800, 1600, and 3200 images to study the relationship with absolute error.

### Statistical analysis

We used the *t* test to compare absolute errors of the surgeons and those of the AI system. Among the AI measurements, absolute errors in surgical patients were compared to errors in non-surgical patients. Stepwise multiple regression analysis was performed with the absolute error at the C2–C7 angle as the response variable and the patients’ demographic data as the explanatory variable. *P* values < 0.05 (two-sided) were considered statistically significant. The error for the AI algorithm was compared with that for Surgeon 1 and that for Surgeon 2. For multiple comparisons, p-values were adjusted using the Bonferroni method. Statistical analysis was performed using the SPSS Statistics software (version 20; IBM, Armonk, NY, USA).

## Results

### Demographic data

A total of 1674 patients with 4546 x-rays were included in our study: 707 males and 967 females (Table 1). The mean age ± standard deviation (SD) was 61 ± 19 years (range, 2–96 years).

**Table 1:** Demographic data of study participants.

Variable	Patients	Cervical X-rays
Number	1674	4546
Men	707	2060
Women	967	2486
Mean age (years)	61 ± 18	N/A
Minimum age (years)	2	N/A
Maximum age (years)	96	N/A
Patients underwent surgery	280	877
Patients did not undergo surgery	1394	3669

N/A, not applicable.

Using the ground truth as a basis, we found the measurements to be –9.4° ± 15.8° (mean ± SD) in the neutral position, 14.3° ± 15.6° in flexion, and –25.1° ± 18.6° in extension (Table 2).

**Table 2:** X-ray Measurements at Each Position Based on the Ground Truth.

Position	Number of x-rays		C2–C7 angle	C2 slope	C7 slope
			(degrees)		
Flexion	1458	Mean ± SD	14.3 ± 15.6	–44.2 ± 14.8	–29.9 ± 11.5
		Max	110.0	11.0	32.7
		97.5th percentile	45.5	−16.0	−8.0
		2.5th percentile	−15.0	−75.0	−52.0
		Min	−36.0	−96.0	−74.7
Neutral	1645	Mean ± SD	–9.4 ± 15.8	–17.2 ± 12.8	–26.6 ± 10.6
		Max	110	34.0	37.0
		97.5th percentile	22.0	4.0	−7.2
		2.5th percentile	−37.0	−47.9	−48.0
		Min	−61.0	−90.0	−67.0
Extension	1443	Mean ± SD	–25.1 ± 18.6	0.33 ± 16.3	–24.8 ± 10.7
		Max	99.6	53.0	35.0
		97.5th percentile	11.0	30.9	−3.1
		2.5th percentile	−58.9	−32.9	−46.0
		Min	−73.0	−92.0	−61.9

SD, standard deviation.

Surgical cases involved 280 participants (17%) with a total of 877 x-rays. In the non-surgical patient, the flexional range of motion (ROM) was 24.4° ± 12.4° (mean ± SD), and the extensional ROM was 14.9° ± 11.5°.

### Absolute error of artificial intelligence relative to ground truth

The MAE of the AI algorithm in all 1674 patients (with a total of 4546 x-rays) was 3.3° ± 4.7° for the C2–C7 angle, 1.7° ± 2.7° for the C2 slope, and 2.7° ± 3.8° for the C7 slope. The median absolute error was 2.2° for the C2–C7 angle, 1.2° for the C2 slope, and 1.7° for the C7 slope. The maximum absolute error was 104.9° for the C2–C7 angle, 63.1° for the C2 slope, and 58.0° for the C7 slope. The AI algorithm took 206 s to measure 4546 x-rays, at an average speed of 0.045 s per x-ray. This algorithm is available at (https://ykszk.github.io/c2c7demo/).

### Relationship between confidence score and absolute error

**Figure 5:**
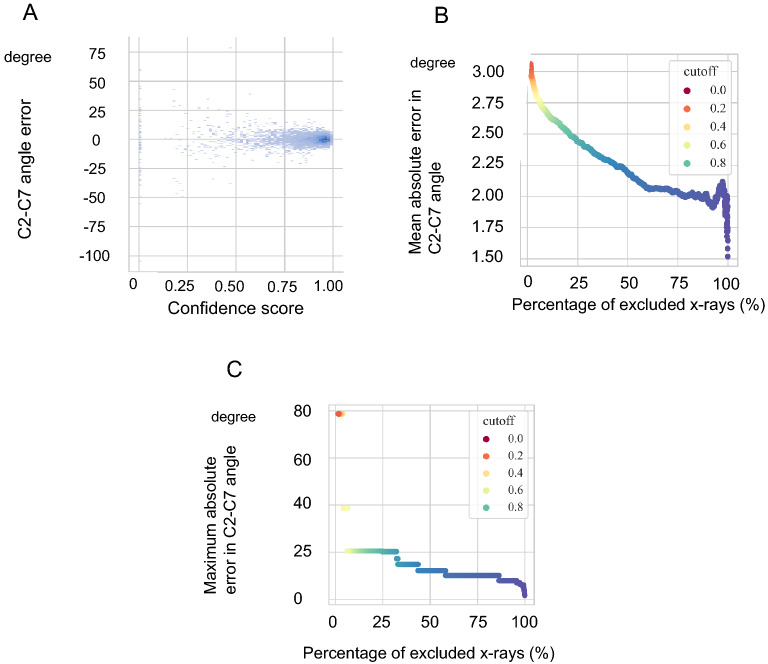
(**A**) Scatter plot showing the relationship between the confidence score and the error at the C2–C7 angle. The smaller the confidence score, the larger the error. (**B**) Relationship between the percentage of excluded x-rays and mean absolute error at the C2–C7 angle when the cutoff value of the confidence score is changed. Increasing the threshold reduces the error but increases the number of x-rays to be excluded. (**C**) Relationship between the percentage of excluded x-rays and maximum absolute error at the C2–C7 angle when the cutoff value of the confidence score is changed.

The mean confidence score was 0.94 ± 0.07 for the C2 slope and 0.88 ± 0.15 for the C7 slope (Fig. 5A). Excluding x-rays with a low confidence score reduced the absolute error. When the threshold was set to 0.6, 309 x-rays (6.8%) were excluded, and the MAE in the C2–C7 angle dropped to 2.7°, the median to 2.1°, and the maximum error to 20.5°. Similarly, when the threshold was set at 0.9, 1909 x-rays (42%) were excluded, and the MAE in the C2–C7 angle dropped to 2.3°, the median to 1.9°, and the maximum error to 14.8° (Figs. 5B,C).

### Artificial intelligence's ability to distinguish between positions taken x-rays

The accuracy of AI to distinguish the positions when x-rays were taken was 98% (Table 3). The mean recall, precision, F-Measure were all 0.983.

**Table 3:** Confusion matrix showing AI’s ability to determine the positions when x-rays were taken.

		Prediction of AI				Prediction indicators				
		Flexion	Neutral	Extension	Total		Recall	Precision	F-Measure	Accuracy
Real positions	Flexion	1437	5	16	1458		0.986	0.980	0.988	0.984
	Neutral	15	1620	10	1645		0.985	0.988	0.987	
	Extension	15	14	1414	1443		0.980	0.982	0.981	
	Total	1467	1639	1440	4546	Mean	0.983	0.983	0.983	

AI, artificial intelligence.

### Comparison of absolute error at the C2–C7 angle for randomly selected participants relative to ground truth between artificial intelligence and surgeons

#### Artificial intelligence

**Figure 6:**
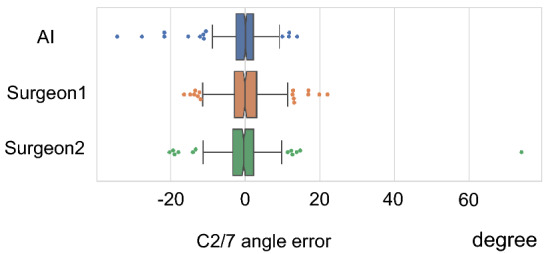
Box plot of the errors at the C2–C7 angle for 168 randomly selected participants with a total of 415 x-rays. The top of the box represents the 75th percentile, the bottom of the box represents the 25th percentile, and the line in the middle represents the 50th percentile. The whiskers represent the highest and lowest values that are not outliers or extreme values. Dots beyond the whiskers represent outliers and extreme values. AI, artificial intelligence.

In the group of randomly selected patients (comprising 168 cases with 415 total x-rays), the MAE of the AI algorithm was 3.1° ± 3.4°, the median absolute error was 2.4°, and the maximum absolute error was 34.3° (Fig. 6 and Table 4).

**Table 4:** Comparison of C2–C7 Angle Errors Between AI and Surgeons for Randomly Selected Cases*

Absolute Error (degree)	AI	Surgeon 1	Surgeon 2	P-value^†^ (AI vs. Surgeons)	
				vs. Surgeon 1	vs. Surgeon 2
Mean	3.1	3.9	3.8	0.002	0.036
Median	2.4	3.0	2.9		
Maximum	34.3	22.1	74.0		
SD	3.4	3.4	4.7		

*168 cases with a total of 415 x-rays. ^†^ The p-values were adjusted by the Bonferroni method.

AI, artificial intelligence; SD, standard deviation.

#### Surgeon 1

For Surgeon 1, the MAE was 3.9° ± 3.4°, the median absolute error was 3.0°, and the maximum absolute error was 22.1°.

#### Surgeon 2

For surgeon 2, the MAE was 3.8° ± 4.7°, the median absolute error was 2.9°, and the maximum absolute error was 74.1°.

### Statistical results

The AI algorithm had a significantly smaller absolute error than Surgeon 1 did (*P* = 0.002) and Surgeon 2 did (*P* = 0.036).

### Relationship between absolute error at the C2–C7 angle for artificial intelligence and background data on participants

A stepwise multiple regression analysis was performed regarding age, sex, whether the patient had undergone surgery, and radiographic posture (flexion, neutral position, extension) as explanatory variables. Being of younger age, being male, and having undergone surgery were related to a larger error rate (Table 5). The MAE for participants who underwent surgery (4.0° ± 6.6°) was significantly larger than for those who did not undergo surgery (3.1° ± 4.1°; *P* < 0.001; Table 6).

**Table 5:** Stepwise multiple regression analysis of absolute error of the AI at the C2–C7 angle as the dependent variable.

Independent Variables	Covariates				
	B	SE	Beta	*t* Test	*P* Value
Age	–0.032	0.004	–0.109	–7.191	< 0.0001
Undergoing surgery	0.935	0.184	0.078	5.089	< 0.0001
Sex (male)	0.321	0.146	0.033	2.196	0.028

The square of the coefficient of multiple correlation (R^2^) in this model = 0.018.

AI, artificial intelligence; B, partial regression coefficient; SE, standard error; beta, standardized partial regression coefficient.

**Table 6:** Comparison of the errors between surgical and nonsurgical cases when measured by artificial intelligence.

Variable	Surgery involved	No Surgery involved	p-value
Number of patients	280	1394	N/A
Number of x-rays	877	3669	N/A
Mean absolute error of C2–C7 angle ± SD (degrees)	4.0 ± 6.6	3.1 ± 4.1	< 0.001

N/A, not applicable; SD, standard deviation.

### Relationship between the absolute error and the number of training images

**Figure 7:**
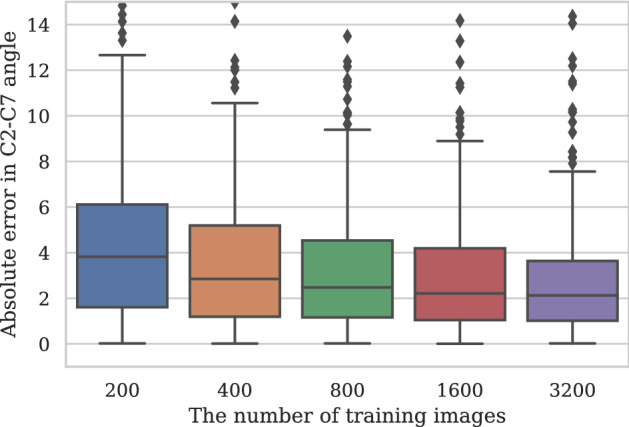
Box plot of the errors at the C2–C7 angle for 168 randomly selected participants with a total of 415 x-rays. The top of the box represents the 75th percentile, the bottom of the box represents the 25th percentile, and the line in the middle represents the 50th percentile. The whiskers represent the highest and lowest values that are not outliers or extreme values. Dots beyond the whiskers represent outliers and extreme values.

The MAE ± SD of the C2-C7 angle was 6.1° ± 11.1° for 200 training images. Similarly, the MAE was 4.7° ± 10.1° for 400 training images, 4.1° ± 10.1° for 800 images, 3.5° ± 4.8° for 1600 images, and 3.3° ± 6.4° for 3200 images (Fig. 7).

### Participants with absolute error of 20° or more at the C2–C7 angle

There were 35 participants with an absolute error of ≥ 20° at the C2–C7 angle. The MAEs for these participants were 35.0° for the C2–C7 angle, 14.2° for the C2 slope, and 23.1° for the C7 slope. The mean confidence score was 0.34. The common reasons for vertebral body misidentification were hardly visible C7 (n = 12; 34%), the presence of severe spine deformity (n = 8; 23%) (Fig. 6), the use of posterior instrumented fusion (n = 7; 20%), the presence of fused vertebrae (n = 6; 17%), and patients being in their infancy (n = 2; 6%).

## Discussion

We created an AI model for 1674 participants with 4546 cervical x-rays. The AI model measured the C2–C7 angle with a MAE of 3.3° and a median absolute error of 2.2°. The AI model can measure the angle quickly with equivalent accuracy to surgeons. Accuracy can be improved by adjusting the confidence score.

To the best of our knowledge, our study is novel in the following respects. First, this is the first study to use AI for automated measurement of cervical lordosis; second, the accuracy is better than in previous studies regarding lumbar spine measurements; third, we introduced a confidence score to measure the AI's confidence. Finally, the AI model has been released as a Graphical User Interface program that anyone can use (https://ykszk.github.io/c2c7demo/). If readers use the graphical user interface program we developed, we believe they will realize its usefulness. This program is Central Processing Unit-driven to make it usable on ordinary computers. Therefore, the measurement takes several seconds, but on a Graphics Processing unit-equipped computer, the measurement is instantaneous.

There have been some earlier reports on automated measurement of the lumbar spine. Cho *et al*.^17^ used AI to measure L1–S1 lumbar lordosis in 780 lumbar spine x-rays from 780 people, excluding those who had undergone surgery, successfully measuring lordosis in 84% of their study participants, with an MAE of 8.055° and a median absolute error of 6.965°. Schwartz *et al*.^16^ used AI to measure L1–S1 lumbar lordosis in 816 lumbar spine x-rays of 816 patients older than age 18 years, including 6.1% who underwent spinal instrumentation. The MAE was 4.3°, and the median absolute error was 2.2°. Korez *et al*.^24^ measured spinopelvic parameters in 55 patients using AI and reported that the MAE ranged from 1.2° to 5.5°.

In general, the cervical spine may be more challenging to measure than the lumbar spine because the shoulder girdle may hide C7. However, our results were better than for previous measurements of the lumbar spine. Several factors may have contributed to the decrease in error, and it isn't easy to pinpoint which one played a major role. An increase in training data and a reduction in processing steps might be possible contributing factors (Fig. 7). Previous researchers manually segmented all vertebrae, extracted the vertebrae, and then measured the angle. However, we directly measured the angle by annotating only the vertebral vertices needed for the angle measurement. This reduced the number of processing steps and might contribute to reducing the absolute error. The reduction in the process made the annotation work task easier. As a result, we could annotate more images, leading to more training data.

**Figure 8:**
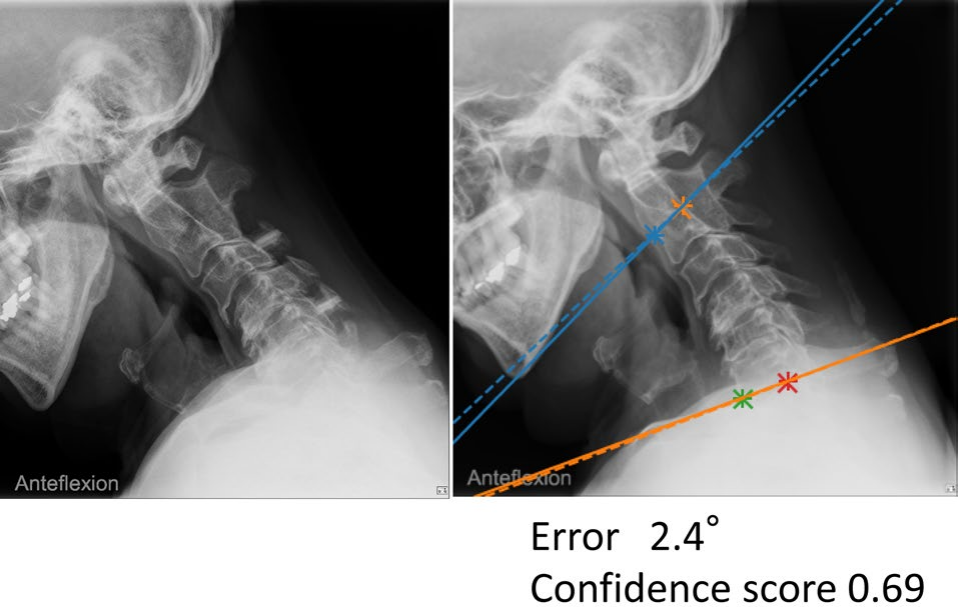
A case of correct measurement despite the presence of fused vertebrae. Left: before measurement. Right: after measurement. The solid lines represent the ground truth, and the dashed lines represent the measurement obtained by artificial intelligence.

One of the limitations of AI in our study was that the maximum absolute error was large. Because we excluded no participants, congenitally fused or malformed vertebrae were included (Fig. 8). Some of the advanced deformities were difficult to measure, even for surgeons (Fig. 3). Initially, we considered excluding such difficult cases because it would be too much to ask AI to measure them. However, we felt that these difficult cases should be included because they are rather valuable as training data. As a result, the maximum error became larger. Influenced by the maximum error, the mean absolute error was slightly larger at 3.3 degrees, but the median error was 2.2 degrees, which is acceptable for measuring the C2-C7 angle of the cervical spine.

Addressing rare cases is a complex problem, but there are two possible ways to manage them. One is data augmentation. Because it is difficult to collect rare cases, data is generated using a generative adversarial network^25^. The other method is to report rare case measurements as outliers. If the AI exhibits an angle at the C2 or C7 slope that would not usually occur, AI should display a warning. For this purpose, an upper and lower limit of the measured value should be set. For example, the minimum value of the C2 slope at the neutral position was −90.0°, but the 2.5th percentile was only −47.9°. If the AI measurement exceeds this 2.5th percentile or 97.5th percentile, the AI will report it as an outlier and recommend human confirmation.

Despite some errors, we can advocate many uses for this AI. For example, incorporating an AI measurement function into an existing image viewer to help in measurement will greatly improve work efficiency. Surgeons do not need to measure manually but only need to check AI measurement lines. If surgeons determine that the measurement is incorrect, they can correct it. As AI learns more and more, it is expected to become more and more accurate.

Researchers can use AI for clinical research. Researchers take a long time to measure manually, and their work efficiency decreases over time because of fatigue. However, AI can take measurements quickly, and there is no such decrease in work efficiency. Researchers can control the error by adjusting the confidence score. The accuracy of the measurement can be assured by stating the set confidence score and the absolute error in the report. For x-rays with low confidence scores and unreliable AI measurements, researchers should measure these x-rays themselves. Although it is difficult to determine a uniform standard, we recommend a threshold of 0.6 for the confidence score; setting it at 0.6, 94.2% of x-rays can be measured, with a mean absolute error of 2.7°. This is better than the human measurement error, which is generally considered to be over 3°^7,8^. In previous clinical research involving x-ray measurements without AI, a researcher needed another researcher to measure and report inter-examiner errors. However, by using AI trained on data agreed upon by multiple researchers, AI may be able to replace the other researcher. This can reduce the human resources needed to conduct the research^10^.

In conclusion, we have successfully developed an AI tool for rapid and accurate automated measurement of cervical x-rays. These tools have a high clinical application value. However, because of the large errors in rare cases such as highly deformed ones, AI may, in principle, be limited to assisting humans.

## Supplementary Information

Supplementary Information.

## Data Availability

All data generated or analysed during this study are included in this published article and its supplementary information files. The X-ray images used to support the findings of this study are available upon request from the corresponding author. The algorithm is available in the repository, [https://github.com/ykszk/c2c7demo].
